# Neurodevelopmental outcomes of school-age children conceived after hysterosalpingography with oil-based or water-based iodinated contrast: long-term follow-up of a nationwide randomized controlled trial

**DOI:** 10.1093/humrep/deae183

**Published:** 2024-08-28

**Authors:** Sarai M Keestra, Nienke Van Welie, Kim Dreyer, Rik Van Eekelen, Tessa J Roseboom, Jaap Oosterlaan, Ben W Mol, Martijn J J Finken, Velja Mijatovic, Marsh Königs

**Affiliations:** Department of Paediatric Endocrinology, Emma Children’s Hospital, Amsterdam UMC, Vrije Universiteit Amsterdam, Emma Children’s Hospital, Amsterdam, The Netherlands; Department of Epidemiology & Data Science, Amsterdam UMC, University of Amsterdam, Amsterdam, The Netherlands; Amsterdam Reproduction & Development Research Institute, Amsterdam UMC, Amsterdam, The Netherlands; Amsterdam Reproduction & Development Research Institute, Amsterdam UMC, Amsterdam, The Netherlands; Department of Reproductive Medicine, Amsterdam UMC, Vrije Universiteit Amsterdam, Amsterdam, The Netherlands; Amsterdam Reproduction & Development Research Institute, Amsterdam UMC, Amsterdam, The Netherlands; Department of Reproductive Medicine, Amsterdam UMC, Vrije Universiteit Amsterdam, Amsterdam, The Netherlands; Department of Epidemiology & Data Science, Amsterdam UMC, University of Amsterdam, Amsterdam, The Netherlands; Department of Epidemiology & Data Science, Amsterdam UMC, University of Amsterdam, Amsterdam, The Netherlands; Amsterdam Reproduction & Development Research Institute, Amsterdam UMC, Amsterdam, The Netherlands; Department of Obstetrics & Gynaecology, Amsterdam UMC, University of Amsterdam, Amsterdam, The Netherlands; Amsterdam Reproduction & Development Research Institute, Amsterdam UMC, Amsterdam, The Netherlands; Emma Neuroscience Group & Follow-Me Program, Emma Children’s Hospital, Department of Pediatrics, Amsterdam UMC, University of Amsterdam, Amsterdam, The Netherlands; Department of Obstetrics & Gynaecology, School of Clinical Sciences, Monash University, Melbourne, Australia; Department of Paediatric Endocrinology, Emma Children’s Hospital, Amsterdam UMC, Vrije Universiteit Amsterdam, Emma Children’s Hospital, Amsterdam, The Netherlands; Amsterdam Reproduction & Development Research Institute, Amsterdam UMC, Amsterdam, The Netherlands; Amsterdam Reproduction & Development Research Institute, Amsterdam UMC, Amsterdam, The Netherlands; Department of Reproductive Medicine, Amsterdam UMC, Vrije Universiteit Amsterdam, Amsterdam, The Netherlands; Amsterdam Reproduction & Development Research Institute, Amsterdam UMC, Amsterdam, The Netherlands; Emma Neuroscience Group & Follow-Me Program, Emma Children’s Hospital, Department of Pediatrics, Amsterdam UMC, University of Amsterdam, Amsterdam, The Netherlands

**Keywords:** iodinated contrast media, hysterosalpingography, HSG, tubal flushing, fertility work-up

## Abstract

**STUDY QUESTION:**

Does preconceptional exposure to oil-based iodinated contrast media during hysterosalpingography (HSG) impact children’s neurodevelopment compared with exposure to water-based alternatives?

**SUMMARY ANSWER:**

Our study found no large-sized effects for neurodevelopment in children with preconceptional exposure to oil-based iodinated contrast media during HSG compared with water-based alternatives.

**WHAT IS KNOWN ALREADY:**

HSG is widely used as a diagnostic tool in the female fertility work-up. Tubal flushing with oil-based iodinated contrast has been shown to enhance fertility outcomes in couples with unexplained infertility, increasing the chances of pregnancy and live birth compared with water-based alternatives. However, oil-based contrast contains higher doses of iodine and has a longer half-life, and concerns exist that iodinated contrast media can affect women’s iodine status and cause temporary (sub)clinical hypothyroidism in mothers and/or foetuses. Considering that thyroid hormones are vital to embryonal and foetal brain development, oil-based contrast media use could increase the risk of impaired neurodevelopment in children conceived shortly after HSG. Here we examine neurodevelopmental outcomes in school-aged children conceived after HSG.

**STUDY DESIGN, SIZE, DURATION:**

This is a long-term follow-up of the H2Oil trial in which oil-based or water-based contrast was used during HSG (Netherlands; 2012–2014; NTR3270). Of 369 children born <6 months after HSG in the study, we contacted the mothers of 140 children who gave consent to be contacted for follow-up. The follow-up study took place from January to July 2022 (NCT05168228).

**PARTICIPANTS/MATERIALS, SETTINGS, METHODS:**

The study included 69 children aged 6–9 years who were conceived after HSG with oil-based (n = 42) or water-based contrast (n = 27). The assessments targeted intelligence (Wechsler Intelligence Scale for Children), neurocognitive outcomes (computerized neurocognitive tests), behavioural functioning (parent and teacher questionnaires), and academic performance. Linear regression models, adjusted for age, sex, and parental educational attainment were employed to compare groups.

**MAIN RESULTS AND THE ROLE OF CHANCE:**

School-aged children born to mothers after oil-based contrast HSG did not significantly differ from children born to mothers after water-based contrast HSG, in regards to intelligence, neurocognitive functioning, behavioural functioning, or academic performance, with the exception of better performance for visuomotor integration functions in children exposed to oil-based contrast preconception. After exploratory correction for multiple comparisons, none of the group differences was statistically significant.

**LIMITATIONS, REASONS FOR CAUTION:**

The small sample size of this follow-up study limited statistical power. This study provides evidence for the absence of large-sized differences between preconceptional exposure to the two contrast media types but does not rule out more subtle effects on neurodevelopment compared to naturally conceived children without preconceptional exposure to HSG.

**WIDER IMPLICATIONS OF THE FINDINGS:**

This study contributes to our knowledge about the long-term effects of different types of iodinated contrast media used in fertility work-up, indicating that choosing oil-based over water-based iodinated contrast media is unlikely to have major effect on the long-term neurodevelopmental outcomes of children conceived shortly after HSG. However, further research should focus on the overall safety of iodine exposure during HSG, comparing children conceived after HSG to those conceived naturally as both types of contrast contain high amounts of iodine.

**STUDY FUNDING/COMPETING INTEREST(S):**

The original H2Oil randomized controlled trial was an investigator-initiated study that was funded by the two academic hospitals now merged into the Amsterdam University Medical Centre. The current follow-up study (Neuro-H2Oil) is funded through a research grant awarded to the authors by the Amsterdam Reproduction & Development (AR&D) research institute. S.K. is funded by a AMC MD/PhD Scholarship from the Amsterdam UMC. S.K. reports holding voluntary roles in the civil society organizations Universities Allied for Essential Medicines and People's Health Movement. V.M. reports receiving travel and speaker fees as well as research grants from Guerbet, Merck and Ferring. K.D. reports receiving travel and speaker fees as well as research grants from Guerbet. BWM is supported by a NHMRC Investigator grant (GNT1176437) and reports consultancy, travel support and research funding from Merck, consultancy for Organon and Norgine, and holding stock from ObsEva. The other authors report no conflict of interest.

**TRIAL REGISTRATION NUMBER:**

NCT05168228

## Introduction

Hysterosalpingography (HSG) is a widely used diagnostic tool for the evaluation of tubal patency in the fertility work-up (Roest *et al.*, 2021). Tubal flushing during HSG using oil-based contrast heightens the likelihood of pregnancy and a subsequent live birth, increases the chance of a naturally conceived pregnancy, and reduces the time to pregnancy compared to water-based alternatives (Dreyer *et al.*, 2017; Wang *et al.*, 2019, 2020; van Rijswijk *et al.*, 2020). This fertility-enhancing effect of oil-based contrast is the most pronounced immediately after HSG and gradually decreases over time (van Welie *et al*., 2020b). However, accumulating evidence suggests that the iodinated contrasts used during HSG, particularly oil-based contrast, may interfere with normal thyroid function (Mekaru *et al.*, 2008; So *et al.*, 2017; Mathews *et al.*, 2022). A recent follow-up study of pregnancies conceived after HSG with oil-based contrast additionally showed that for half of the women, the iodine concentration remains elevated throughout pregnancy, and iodine excess is found in mother's milk and neonatal urine postpartum (Li *et al.*, 2021). Therefore, significant concerns exist regarding the long-term effects of the high iodine content of the contrast media used in HSG on the development of offspring conceived shortly after the procedure (Li *et al.*, 2021; Mathews *et al.*, 2021; van Welie *et al.*, 2021).

Iodinated contrast media used in HSG can affect women's iodine status and thyroid function (Mekaru *et al.*, 2008; Kaneshige *et al.*, 2015; So *et al.*, 2017; Li *et al.*, 2021; Mathews *et al.*, 2021, 2022). After exposure to high levels of iodine, a transient suppression of thyroid function may occur, which is known as the Wolff–Chaikoff effect (Wolff and Chaikoff, 1948; Theodoropoulos *et al.*, 1979; Lee *et al.*, 2015; Mathews *et al.*, 2021). This autoregulatory mechanism temporarily reduces thyroid hormone synthesis but is usually self-limiting; thyroid hormone production is resumed when iodine levels have returned to below a critical threshold (Wolff and Chaikoff, 1948; Leung and Braverman, 2014; de Carmo Campos *et al.*, 2023). Women are at a significant risk of displaying subclinical hypothyroidism and isolated low thyroxine levels (hypothyroxinaemia) in the context of iodine excess in the first weeks of pregnancy (Shi *et al.*, 2015). Oil-based contrast contains higher doses of iodine (480 mg/ml) compared to water-based contrast (ranging 240–300 mg/ml) (van Welie *et al.*, 2020a; Mathews *et al.*, 2021). While safety and pharmacokinetics studies in animals show that water-based contrast is eliminated within two days, oil-based contrast can persist for as long as fifty days, leading to an increased iodine concentration in the thyroid (Miyamoto *et al.*, 1995). Recent clinical studies show that HSG using oil-based contrast can cause iodine excess resulting in temporary (sub)clinical hypothyroidism in women shortly after the procedure (Mekaru *et al.*, 2008; Kaneshige *et al.*, 2015; So *et al.*, 2017; Li *et al.*, 2021; Mathews *et al.*, 2021, 2022). The suppressed maternal thyroid function under the Wolff–Chaikoff effect may temporarily hinder the 50% rise in thyroid hormone production that normally occurs during early pregnancy when the offspring does not yet produce its own thyroid hormones (Haddow *et al.*, 1999; Pop *et al.*, 1999; Glinoer, 2007; Lee *et al.*, 2015; Thompson *et al.*, 2018). Iodine in contrast media can also to enter the foetal compartment, where it may affect foetal thyroid function itself (Eng *et al.*, 1999; Moon *et al.*, 2000; Vanhaesebrouck *et al.*, 2005). The foetal thyroid is particularly vulnerable to the Wolff–Chaikoff effect until late gestation (Eng *et al.*, 1999; Lee *et al.*, 2015; Alexander *et al.*, 2017; Hardley et al., 2018), and animal studies show that iodine can accumulate in the amniotic fluid (Book *et al.*, 1974). Consequently, if conception takes place shortly after HSG, iodine in contrast media has the potential to impact maternal as well as foetal thyroid function.

Due to the absence of a functional foetal thyroid gland until the second trimester of pregnancy, early in gestation, the offspring completely relies on the maternal thyroid function during a crucial developmental period (Moon *et al.*, 2000; Vanhaesebrouck *et al.*, 2005). Animal and human studies demonstrate that an adequate supply of maternal thyroid hormones is vital to embryonal and foetal brain development (Pharoah *et al.*, 1971; Lazarus, 1999; Lavado-Autric *et al.*, 2003; Ausó *et al.*, 2004; Zoeller and Rovet, 2004). Even mild inadequacy of the thyroid axis during early gestation may result in negative effects on the child’s intelligence and psychomotor development (Pop *et al.*, 1999; Li *et al.*, 2010; Korevaar *et al.*, 2018; Thompson *et al.*, 2018), slower processing speed during reaction time tests (Finken *et al.*, 2013), with heightened risk of expressive language delay (Henrichs *et al.*, 2010), suboptimal performance on arithmetic tests (Noten *et al.*, 2015), and greater scores on symptoms of attention deficit disorder (Modesto *et al.*, 2015), albeit not unequivocally (Thompson *et al.*, 2018). These findings raise concerns regarding the potential impact of iodine exposure and subsequent disturbance of maternal and/or foetal thyroid function on foetal development if conception takes place shortly after HSG.

To address concerns regarding the potential impact of iodinated contrast media on the offspring's thyroid axis, we previously investigated thyroid function in Dutch neonates born after HSG (van Welie *et al.*, 2020a). We did not observe an increase in congenital hypothyroidism in children conceived after HSG with either type of contrast (van Welie *et al.*, 2020a), as opposed to findings from Japan, which is known for its iodine-rich diets and a higher prevalence of congenital hypothyroidism (Satoh *et al.*, 2015). The absence of neonatal thyroid dysfunction after conception with HSG was reassuring, but did not rule out the possibility that nascent brain development is perturbed by a temporary decrease in thyroid hormone availability that can no longer be detected postnatally. Although it is not possible to test foetal thyroid function in utero non-invasively, it is possible to study the potential long-term consequences of preconceptional exposure to iodine excess by assessing the neurodevelopment of exposed offspring.

With more than 5000 HSGs performed annually in the Netherlands alone (Roest *et al.*, 2020) and studies reporting the effects of HSG on thyroid function particularly after the use of oil-based contrast (Mekaru *et al.*, 2008; Kaneshige *et al.*, 2015; So *et al.*, 2017; Mathews *et al.*, 2022), the safety of iodinated contrast media the neurodevelopment of offspring conceived shortly after HSG warrants investigation. To optimally inform parents about the possible iatrogenic effect of HSG on offspring development, a comprehensive assessment of the long-term neurodevelopmental outcomes of children conceived after HSG is needed. In the current study, we present the first long-term follow up examining the neurodevelopment of children aged 6–9 years who were conceived within 6 months after HSG with oil- or water-based contrast, as part of a nationwide multicentre randomized controlled trial (Dreyer *et al.*, 2017). We aimed to determine whether the use of oil-based contrast, with its prolonged and higher iodine exposure, poses a greater risk for abnormal neurodevelopment in school-aged children, as compared to water-based alternatives in HSG. This would provide better information for future parents and healthcare professionals about the safety considerations associated with choosing one or the other contrast type during this widely employed diagnostic technique.

## Materials and methods

### Study design and ethics

**Figure 1.**
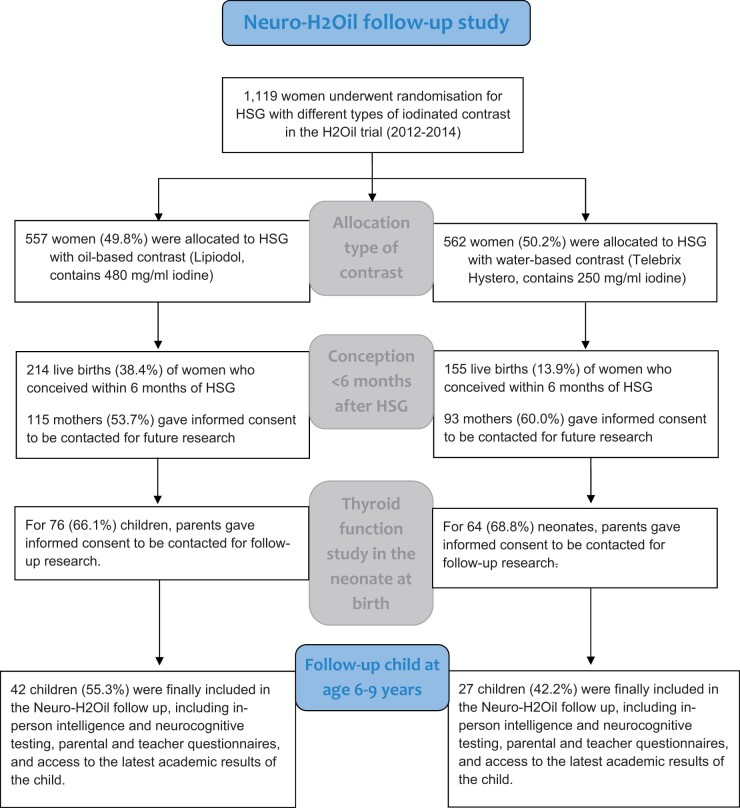
**Flowchart of the Neuro-H2Oil follow-up study (2021-2022)**.

The H2Oil trial was a nationwide multicentre (27 hospitals) randomized controlled trial (RCT) conducted between 2012 and 2014 to assess the impact of tubal flushing during HSG with oil-based versus water-based contrast on fertility outcomes in couples with unexplained or mild male infertility (Fig. 1). A total of 1119 women were originally randomly assigned to receive HSG with either water-based or oil-based iodinated contrast, characterized by differences in iodine content (480 mg/ml for oil- and 240–300 mg/ml for water-based contrast). Among these women, 369 conceived a child within 6 months after HSG in the context of the H2Oil trial, of whom 208 (56.3%) provided informed consent to be contacted for future research. In a previous study of these children born from the H2Oil trial, which focused on evaluating neonatal thyroid function (van Welie *et al.*, 2021), the mothers of 140 (67.3%) children gave consent to be contacted for follow-up studies in their children. Among this group, 76 women had received HSG with oil-based contrast and 64 women had received water-based contrast. Prior to participant recruitment, the current follow-up study (Neuro-H2Oil) was prospectively registered on ClinicalTrials.Gov (NCT05168228). Ethical approval for the study protocol was granted by the medical research ethics committee of Amsterdam UMC, location VU Medical Centre (2021.0448).

### Inclusion and exclusion criteria

The present follow-up study included children who met the following criteria: (i) conception occurred in the 6 months after the mother underwent HSG with either oil-based or water-based contrast as part of the H2Oil trial, and (ii) maternal consent for further follow-up research was obtained at time of participation to the original H2Oil trial. Children were excluded from the study if they met any of the following criteria: (i) documented and/or parent-reported diagnosis of a neurological disorder, (ii) presence of severe motor disability that impeded the assessment of outcomes, (iii) severe somatic disorders known to impact the outcome assessments at the time of evaluation (iv) inability to comprehend the instructions provided during the assessment, or (v) withdrawal of informed consent by their parents during the course of the study.

### Recruitment of participants

All mothers of the initially identified 140 children were contacted via postal mail, for non-responding parents additional recruitment attempts were made through phone calls or emails. Out of these efforts, the parents of 72 (51.4%) children expressed their willingness to participate with their child in the follow-up study. However, during the screening process, two children were subsequently excluded from the analysis due to documented cases of congenital abnormalities that were considered to be unlikely to be related to the HSG procedure. Additionally, one child was excluded due to autism spectrum disorder, which would have rendered the child unable to comprehend the testing instructions during the assessment. Consequently, the final cohort for analysis consisted of 69 children (Fig. 1). We compared the demographic characteristics of the mothers of the children in the Neuro-H2Oil follow-up study with the characteristics of the overall H2Oil trial participants to account for possible attrition bias.

### Study procedure

Following the initial recruitment phase, children and their parents were invited to one of the hospitals that participated in the original H2Oil study, or they were visited at home for comprehensive in-person neurocognitive testing. The aim was to ensure convenience and accessibility for all participants to minimize loss to follow-up. During the assessments, children underwent a series of neurocognitive tests. Parents were asked to complete a questionnaire concerning their child's medical history and family situation. In addition, parents and teachers were provided with questionnaires to assess the child's behavioural functioning. With parental permission, schools were kindly requested to share the latest academic performance of the participating children.

### Participant characteristics

Demographic characteristics (sex and age of the child) were derived from the original H2Oil study records. Educational achievement of both parents was self-reported on an eight-point scale using a custom questionnaire based on the Dutch Central Bureau of Statistics’ version of the International Standard Classification of Education (ISCED), with higher scores indicating higher educational levels (Pleijers and de Vries, 2021). Medical history of the child was taken using a parental questionnaire, assessing whether the child had received a diagnosis of a neurological disorder, ADHD, a learning disability, a language or speech disorder or a thyroid problem, or ever had to take thyroid medication.

### Primary outcome measurements

#### Intelligence

General intelligence was assessed using a shortened Fifth version of the Wechsler Intelligence Scale for Children (WISC-V) (Hurks *et al.*, 2016). Full-scale IQ (FSIQ) was estimated using the subtests Vocabulary, Similarities, Matrix Reasoning, and Block Design (Zhou and Raiford, 2011; Hurks *et al.*, 2016; Raiford *et al.*, 2016). Full-scale IQ (FSIQ) estimated with this short form has good validity (*r* = 0.82) and excellent reliability (*r* = 0.92) (Sattler, 2008a,b). All IQ forms were rated by at least two independent assessors.

#### Behavioural functioning

Behavioural functioning was measured through the Dutch version of the Strengths and Difficulties Questionnaire (SDQ), a widely used tool to assess symptoms of the main behavioural and emotional disorder symptoms in children (Van Widenfelt *et al.*, 2003; Brann *et al.*, 2018). The SDQ consists of 25 items divided over five scales: Emotional Symptoms, Conduct Problems, Hyperactivity/Inattention, Peer Problems, and Pro-Social Behaviour (Van Widenfelt *et al.*, 2003; Hall *et al.*, 2019). These scales can be combined to measure the presence of both internalizing disorders symptoms (Internalizing scale) and externalizing disorders symptoms (Externalizing scale). The Strengths and Weaknesses of ADHD Symptoms And Normal behaviour (SWAN) scale (Swanson *et al.*, 2012) provides insight into Attention Deficit Hyperactivity Disorder (ADHD) symptoms in particular (American Psychological Association (APA), 1994). The SWAN scale allows for the differentiation between symptoms of ADHD inattentive and hyperactive/impulsive subtype symptoms (Swanson *et al.*, 2012). The SDQ questionnaire and SWAN scale was given out to parents as well as teachers of the participating children. Validity and reliability of both questionnaires have been established (Swanson *et al.*, 2012; Brann *et al.*, 2018).

#### Academic performance

Academic performance was assessed using standardized tests from the pupil monitoring system developed by the Dutch National Institute for Educational Measurement (CITO) (Gillijns and Verhoeven, 1992). The pupil monitoring system assesses children’s reading, spelling, and arithmetic performance in grades 1–8 in 90% of primary schools in the Netherlands (Janssen *et al.*, 2015; Krom *et al.,* 2010; Tomesen *et al.*, 2015), and are tested for reliability and validity (Janssen *et al.*, 2015; Tomesen *et al.*, 2015). We requested the parents and schools of the participants to share the latest test results of the pupil monitoring system. Age-corrected *z*-scores were used, reflecting the child's academic performance as compared to a standardized norm population as provided by the Dutch National Institute for Educational Measurement.

### Secondary outcome measurements

#### Neurocognitive functioning

Neurocognitive functioning was measured using the ‘Emma Toolbox for Neurocognitive Functioning’, an in-house developed composition of computerized tests based on well-known neuroscientific paradigms with established validity and reliability (Königs *et al.*, 2021). The Emma Toolbox assesses the following neurocognitive domains: processing and control (measuring the speed, stability and consistency of information processing, and interference control), verbal memory (measuring the encoding and consolidation of verbal information in long-term memory), visual memory (measuring the encoding and consolidation of visual information in long-term memory), verbal working memory (measuring encoding and manipulation of verbal information in short-term memory), visual working memory (measuring encoding and manipulation of visual information in short-term memory), and visuomotor integration (measuring the integration of predictable and unpredictable visual information with motor response). Scores are expressed as *z*-scores, where higher scores reflect better neurocognitive performance. See Supplementary Table S1 for detailed descriptions of the neurocognitive tests used (Königs *et al.*, 2021). The Emma Toolbox is programmed in Python using OpenSesame Software and administered on a 15-inch laptop from a 50 cm viewing distance (Mathôt *et al.*, 2012).

### Statistical analysis

To detect effects of clinical relevance, we aimed for a sample size that would allow us to detect medium-sized effects (Cohen’s *d* = 0.5, power of 80% and two-sided alpha = 0.05), as a result of which we aimed to recruit at least 64 children per group. Data were collected in Castor and analyzed in R version 4.0.3 (R Core Team, 2014; Castor EDC, 2019). Missing data (<15%) were imputed using the MICE package; details can be found in Supplementary Table S2. To compare the outcomes between the groups (oil-based contrast group and water-based contrast group), we employed linear regression analysis with group as the independent variable. The analyses were adjusted for age and sex of the child as well as parental educational attainment, by adding these covariates to the regression models.

## Results

This study encompassed a total of 69 children between the ages of 6 and 9 years. Detailed characteristics of the groups are displayed in Table 1. There were no significant differences between the oil-based (n = 42) and water-based (n = 27) groups in terms of key demographic factors such as the age and sex of the child and educational attainment of the parents (Table 1). Additionally, there were no significant differences between the two groups regarding gestational age at birth (Table 1), complications during the pregnancy or postpartum period, or incidence of congenital abnormalities (Supplementary Table S3).

**Table 1.** Demographic characteristics of the participants of the Neuro-H2Oil study.

	Oil-based contrast	Water-based contrast	Overall Neuro-H2Oil Study Sample
n	42	27	69
**Demographic characteristics**			
Girls, n (%)	19 (45.2%)	17 (63.0%)	36 (52.2%)
Age at assessment in y mean (SD)	7.65 (0.72)	7.69 (0.77)	7.67 (0.74)
Parental educational attainment, mean (SD)	5.89 (0.79)	6.09 (0.85)	5.97 (0.81)
**Characteristics conception and pregnancy**			
Duration subfertility in months, mean (SD)	20.5 (9.8)	20.6 (10.5)	20.6 (10.0)
Days between HSG and conception, mean (SD)	75.76 (56.47)	73.37 (51.70)	74.83 (54.28)
Conceived after further IVF treatment, n (%)	1 (2.4%)	0 (0)	1 (1.4%)
Smoking during pregnancy, n (%)	4 (9.5%)	1 (3.7%)	5 (7.2%)
Gestational age at birth in weeks, mean (SD)	39.69 ± 1.52	39.26 ± 1.58	39.52 ± 1.55
**Parent-reported diagnosed medical conditions in the child**			
ADHD, n (%)	3 (7.1%)	0 (0)	3 (4.3%)
Learning disorder, n (%)	1 (2.4%)	0 (0)	1 (1.4%)
Language/speech disorder, n (%)	3 (7.1%)	0 (0)	3 (4.3%)
Hypothyroidism	0 (0)	0 (0)	0 (0)
Hyperthyroidism	0 (0)	0 (0)	0 (0)

n, number; ADHD, attention-deficit hyperactivity disorder.

We did not reach our recruitment target of 64 children per exposure group; therefore, the analyses were powered for moderately- to large-sized effects only (Cohen's *d* = 0.78). To assess the representativeness of the current sample to the original sample participating in the H2Oil trial, we compared characteristics of the mothers in this follow-up study with those in the original trial and found no differences in terms of age, BMI, ethnicity and smoking status (Supplementary Table S4).

Comparisons between the oil-based and water-based contrast groups (Table 2, Supplementary Table S5) revealed no significant differences in primary outcomes, such as intelligence (IQ) behavioural functioning (SDQ: internalizing and externalizing behaviour problems, SWAN: ADHD inattentive and hyperactive/impulsive symptoms) or academic performance (arithmetic, spelling, or technical reading performance). For the secondary outcomes, we did observe a statistically significant difference in favour of the oil-based contrast group for visuomotor integration performance when compared to the water-based contrast group, but there were no differences in other neurocognitive domains.

**Table 2.** The neurodevelopmental outcomes of children conceived after oil- versus water-based iodinated contrast media use during HSG, corrected for age, sex and parental educational attainment.

	Oil-based contrast mean (SD)	Water-based contrast mean (SD)	Adjusted mean difference (95% confidence interval)^1^
n	42	27	69
**Intelligence**			
IQ	105.33 (11.23)	102.78 (12.61)	4.09 (−1.53; 9.71)
**Behavioural outcomes—parent reported**			
Internalizing Problems	3.76 (3.31)	2.78 (2.93)	0.82 (−0.76; 2.41)
Externalizing Problems	5.05 (4.67)	3.96 (2.86)	0.68 (−1.37; 2.73)
Attention problems	−0.34 (0.95)	−0.46(0.68)	0.08 (−0.37; 0.52)
Hyperactivity problems	−0.26 (1.01)	−0.17 (0.65)	−0.16 (−0.61; 0.29)
**Behavioural outcomes—teacher reported**			
Internalizing Problems	1.90 (2.13)	2.33 (2.71)	−0.25 (−1.43; 0.92)
Externalizing Problems	2.75 (2.95)	3.22 (2.76)	−0.88 (−2.29; 0.53)
Attention problems	−0.58(0.89)	−0.48(0.73)	−0.14 (−0.55; 0.26)
Hyperactivity problems	−0.53(0.97)	−0.55(0.93)	−0.06 (−0.52; 0.39)
**School performance** ^2^			
Arithmetic	0.74 (0.93)	0.31 (0.82)	0.43 (−0.14; 1.04)
Spelling	0.56 (0.88)	0.64 (0.99)	−0.10 (−0.73; 0.52)
Technical reading	0.28 (0.99)	0.02 (1.02)	0.21 (−0.43; 0.86)
**Neurocognitive outcomes—domains**			
Visuomotor Integration	**0.19 (0.89)**	−**0.31 (1.10)**	**0.46 (0.04; 0.89)**
Processing & Control	−0.01 (1.03)	0.02 (0.97)	0.03 (−0.42; 0.48)
Visual Memory	0.12 (1.00)	−0.19 (0.98)	0.22 (−0.69; 0.62)
Verbal Memory	−0.05 (1.02)	0.08 (0.98)	−0.17 (−0.65; 0.31)
Visual Working Memory	0.09 (1.01)	−0.15 (0.99)	0.25 (−0.22; 0.71)
Verbal Working Memory	0.00 (1.00)	0.00 (1.02)	0.12 (−0.31; 0.54)

Results for which *P* < 0.05 are shown in bold.

1 Adjusted for parental education, sex, and age of the participant. Uncorrected mean differences and *P*-values are displayed in Supplementary Table S5.

2 Academic performance data are not imputed because >15% of the data are missing. The sample size for the academic performance of the oil-based contrast group is n = 32, and that of the water-based contrast group is n = 14. Supplementary Table S2 contains further information on missing data.

IQ, intelligence quotient.

## Discussion

This is the first study to investigate the long-term consequences of using iodinated contrast media during HSG on the neurodevelopment of offspring conceived shortly after HSG. This follow-up cohort of an RCT involved 69 children conceived within 6 months of HSG with two different types of contrast that differ in iodine content. We did not find differences in neurodevelopmental outcomes in children conceived after HSG with oil-based contrast compared to water-based contrast, except for slightly better performance in the oil-based contrast group on the visuomotor integration domain during the neurocognitive assessment. We found no evidence for large-sized effects on neurodevelopmental outcomes at a school-age in children conceived after oil-based contrast medium in comparison with peers conceived after water-based contrast use. Considering the modest sample size and limited statistical power, more subtle small-to-moderate effects of contrast type cannot be ruled out.

We compared children aged 6–9 years conceived after HSG with oil-based or water-based contrasts, as oil-based contrast has a higher iodine content (Dreyer *et al.*, 2017), takes longer to clear from the body according to animal models (Miyamoto *et al.*, 1995), and is known to transiently affect women's thyroid function after HSG (Mathews *et al.*, 2022). We did not find any evidence suggesting that greater iodine exposure with oil-based contrast use as compared to iodine exposure with water-based contrast leads to worse neurodevelopmental outcome in school-aged children. More specifically, no adverse effects were found in terms of intelligence, neurocognitive functioning, behaviour or academic performance. We did observe that children conceived after oil-based contrast have better visuomotor integration performance than children conceived after water-based contrast. This unexpected observation may be the result of a chance finding, as after exploratory false-discovery rate (FDR) correction for multiple testing, this difference was no longer significant.

Although it is clinically relevant for clinicians and patients to know whether HSG with water-based iodinated contrast is preferable over HSG with oil-based contrast based on its potential to disrupt thyroid function and subsequent child neurodevelopment, the present study cannot establish that there is no adverse impact of iodine exposure in itself on neurodevelopmental outcomes. Both types of iodinated contrast media used during HSG contain iodine doses that greatly exceed the recommended daily intake for women of reproductive age (150 µg) or pregnant women (250 µg) set by the World Health Organization (WHO and UNICEF, 2007). A single HSG exposes a woman to at least 960 000 µg iodine in the case of oil-based contrast, and 600 000 µg in the case of water-based contrast, assuming ∼2 ml of contrast fluid is retained in the abdomen following the procedure (Mathews *et al.*, 2021). Although the safe upper limit of iodine exposure in early pregnancy remains unclear, as more studies in this area are needed (Shi *et al.*, 2015), iodinated contrast media used in HSG greatly exceeds the current European tolerable upper intake limit of 600 µg/day in pregnancy if conception occurs shortly after the procedure (Scientific Committee on Food, 2002). Comparing oil- with water-based contrast exposure therefore cannot preclude the possibility that both types of contrast media have a detrimental effect on child neurodevelopment while the difference in impact between the two contrasts is perhaps negligible, as they both contain an iodine dose per ml of contrast fluid that is more than a thousand times higher than the recommended daily dose (WHO and UNICEF, 2007). Children conceived after water-based contrast exposure, which also contains high levels of iodine, are therefore not an ideal comparison group to establish the overall safety of iodinated contrasts in the periconception period on offspring neurodevelopment. For this reason, we underline the importance of studies comparing neurodevelopmental outcomes of children conceived with HSG to negative controls, i.e. a community sample of naturally conceived pregnancies without HSG, or children from mothers who received a hysterosalpingo-foam sonography (HyFoSy) and therefore not exposed to iodinated contrast preconception.

There is no previous work on the long-term effect of iodine excess due to contrast administration as part of HSG procedure or even radiodiagnostic imaging on neurodevelopmental outcomes of children at a school-age. Existing studies have either focused on the effect of maternal iodine deficiency during pregnancy on neurodevelopment of the offspring, or on the impact of maternal iodine excess on neonatal thyroid function (De Vasconcellos Thomas and Collett-Solberg, 2009; Connelly *et al.*, 2012; van Welie *et al.*, 2020a). The upper acceptable limit of iodine during pregnancy for optimal neurodevelopment therefore remains uncertain (Farebrother *et al.*, 2019), although overuse of dietary supplements containing iodine during pregnancy has been associated with negative effects on motor and cognitive performance in infants (Rebagliato *et al.*, 2013). Overall, there seems to be a U-shaped relationship where both too little and too much exposure to iodine during pregnancy can lead to isolated hypothyroxinaemia and subclinical hypothyroidism in the mother (Shi *et al.*, 2015), and both too high and too low maternal thyroid hormone levels during early pregnancy can negatively affect offspring neurodevelopment (Korevaar *et al.*, 2016). In light of the potential of iodine excess to cause transient thyroid dysfunction in the periconception period, it is important to consider if it is possible to reduce the iodine concentration of oil-based contrast media, or minimize the volume of contrast fluid used during a HSG procedure as much as possible, as a precautionary measure. Additionally, future research should compare HSG-conceived children to peers who have not been exposed to iodine excess in early development. Furthermore, considering the Wolff-Chaikoff effect on the thyroid is transient, it should be established whether timing of conception in relation to date of HSG matters for the offspring’s developmental outcomes, and whether it would be prudent to wait with attempting to conceive until the effect of iodine on thyroid function wears off. For this reason, thyroid function monitoring in the six months following HSG has been recommended (Mathews *et al.*, 2022).

This follow-up study offers several limitations and strengths. Firstly, the original H2Oil trial did not measure thyroid function or iodine status of women before and after HSG, hindering a more in-depth analysis of the impact of different types of iodinated contrast exposure on the maternal thyroid in the Dutch context. Secondly, limited demographic characteristics of the mothers participating in the H2Oil trial were collected. Therefore, we cannot exclude the possibility that attrition may have led to selection bias, although the available information did not reveal evidence suggesting selective attrition, nor did we find evidence for group differences in gestational age, congenital abnormalities, or pregnancy or postpartum complications between the groups. Finally, given the modest sample size, this study was not adequately powered for the detection of small- to medium-sized differences in neurodevelopment. As a result, we currently cannot exclude the possibility that more subtle effects of iodinated contrast type on neurodevelopment exist, for which the detection would require a larger follow-up cohort. We attempted to mitigate the loss to follow-up related to study location and travel time by offering parents the option to conduct neurocognitive testing at home or at a nearby hospital. However, as this study was conducted during the coronavirus pandemic, there was some reluctance of parents to participate in this follow-up. One strength of our study was that the recruitment of the original participants (mothers) was conducted within the context of a RCT, providing a compelling opportunity to investigate the impact of contrast media types on the neurodevelopment of offspring conceived within six months after HSG. Additionally, we followed up on the development of the children nearly a decade after the trial started, and assessed a wide range of neurodevelopmental outcomes spanning domains of functioning with strong relevance to daily life functioning. For future investigations, we recommend recruiting larger samples that would allow the detection of more subtle effects on neurodevelopmental outcomes of children conceived after HSG. We also recommend using an extended follow-up time as some more subtle neurodevelopmental differences may only show later in development.

## Conclusion

In this comprehensive long-term follow-up of children conceived within half a year after their mothers underwent HSG, we found no differences in neurodevelopment in children whose mothers had received oil-based contrast compared to those whose mothers received water-based contrast. Based on these results, there is currently no reason to favour water-based contrast in HSG over oil-based contrast. However, to further evaluate the safety of HSG with iodinated contrast media, we recommend that children conceived after HSG are compared to naturally conceived peers. Replication of this study in larger cohorts is warranted to provide more definitive evidence on the safety of iodinated contrast media use during HSG in relation to child neurodevelopment.

## Supplementary Material

deae183_Supplementary_Table_S1

deae183_Supplementary_Table_S2

deae183_Supplementary_Table_S3

deae183_Supplementary_Table_S4

deae183_Supplementary_Table_S5

## Data Availability

Summary data will be made available on ClinicalTrials.Gov under registration NCT05168228. Individual participant data are available upon request to the corresponding author and subject to consent of the parents.
