# Supplementary material for: Neurodevelopmental outcomes of school-age children conceived after hysterosalpingography with oil-based or water-based iodinated contrast: long-term follow-up of a nationwide randomized controlled trial
Source: Hum Reprod. 2024 Aug 28;39(10):2287–96. doi: 10.1093/humrep/deae183 (PMC11447066; doi:10.1093/humrep/deae183)
Supplement: deae183_Supplementary_Table_S1 [file deae183_supplementary_table_s1.pdf]

Human Reproduction, 2024, 1
https://doi.org/10.1093/humrep/deae183
Supplementary Data

Supplementary Table S1. An overview of the neurocognitive domains tested in the Emma Toolbox for Neurocognitive Functioning.

Neurocognitive domain	Type of task	Explanation of the task	References
Visuomotor integration	Track and trace task	The participant is presented with a moving target stimulus (caterpillar) on the screen and instructed to keep the mouse cursor on the centre of the target in a structured condition (predictable, circular path) and in an unstructured condition (unpredictable, random path) at four linearly increasing target speeds. We measured cursor with a gaming mouse (refresh rate 1000 Hz), correcting the speed of the moving stimulus is for the system refreshing rate.	(De Kieviet *et al.*, 2013)
Processing and control	Attention network test	The participant is presented with target stimuli (airplanes) pointing left or right and instructed to respond as quickly as possible to the direction of a target stimulus by pressing the corresponding button. Performance is influenced by the presentation of cues (central, spatial) and manipulation of target flanker congruency (neutral, congruent, incongruent). We corrected the measurement of reaction times for system latency.	(Fan *et al.*, 2002)
Visual memory	Location learning test	The participant is presented with a grid (5 × 5) with 10 semi-randomly placed visual stimuli five times. After each time this template is shown, the participant has to reproduce it by replacing each visual stimulus in an empty grid, directly after each presentation (direct recall) and after a 15-min interval (delayed recall). Additionally, the participant has to recognize the correct location of each visual stimulus in the grid, among 10 distractor locations (recognition). Displacement is the sum of the differences between the recalled location and the target location expressed in the distance between grid coordinates.	(Bucks and Willison, 1996)
Verbal memory	Rey auditory Verbal learning test (Dutch version)	The participant is presented auditorily with a list of 15 words, which is repeated five times. The subject has to reproduce as many words as possible directly after each presentation (direct recall) and after an interval of 15 min (delayed recall). Lastly, the subject has to select the presented words among 15 distractors (recognition).	(Saan and Deelman, 1986)
Visual working memory	Klingberg task	The participant is presented with a sequence of stimuli (moles) popping up on a four-by-four digital grid (of molehills). Participants are required to repeat the sequence in the order of presentation (forward) or reversed order (backward) by clicking on the relevant locations in the grid. The difficulty increases every other trial, by increasing the length of the sequence or increasing the difficulty of the virtual trajectory of the yellow dots. Performance in each condition is defined by the span (the difficulty level of the last correct trial) multiplied by the stability (the total number of correct trials).	(Nutley *et al.*, 2009)
Verbal working memory	Digit span	The participant is required to repeat a sequence of numbers presented auditorily in the order of presentation (forward condition) or reversed order (backward condition). The difficulty increases every other trial, by increasing the length of the sequence of digits. Performance in each condition is defined by the span (the difficulty level of the last correct trial) multiplied by the stability (the total number of correct trials).	(Wechsler, 1997)

Adapted from Königs *et al.* (2021).

Bucks RS, Willison JR. Development and validation of the location learning test (LLT): a test of visuo-spatial learning designed for use with older adults and in dementia. 1996;**11**:273–286. http://dx.doi.org/101080/13854049708400456 [Internet]
Fan J, McCandliss BD, Sommer T, Raz A, Posner MI. Testing the efficiency and independence of attentional networks. *J Cognit Neurosci* 2002;**14**:340–347.
Kieviet JF De, Stoof CJJ, Geldof CJA, Smits N, Piek JP, Lafeber HN, Elburg RM Van, Oosterlaan J. The crucial role of the predictability of motor response in visuomotor deficits in very preterm children at school age. *Dev Med Child Neurol* 2013;**55**:624–630.
Königs M, Verhoog EM, Oosterlaan J. Exploring the neurocognome: neurocognitive network organization in healthy young adults. *Cortex* 2021;**143**:12–28.
Nutley SB, Söderqvist S, Bryde S, Humphreys K, Klingberg T. Measuring working memory capacity with greater precision in the lower capacity ranges. 2009;**35**:81–95. https://doi.org/101080/87565640903325741 [Internet]
Saan R, Deelman B. Nieuwe 15-Woorden test A en B, 15-WT A en 15-WT B. 1986.
Wechsler D. *WAIS-III: Wechsler Adult Intelligence Scale*. San Antonio, TX: Psychological Corporation, 1997.
