# Supplementary material for: Neurodevelopmental outcomes of school-age children conceived after hysterosalpingography with oil-based or water-based iodinated contrast: long-term follow-up of a nationwide randomized controlled trial
Source: Hum Reprod. 2024 Aug 28;39(10):2287–96. doi: 10.1093/humrep/deae183 (PMC11447066; doi:10.1093/humrep/deae183)
Supplement: deae183_Supplementary_Table_S2 [file deae183_supplementary_table_s2.pdf]

Human Reproduction, 2024, 1
https://doi.org/10.1093/humrep/deae183
Supplementary Data

Supplementary Table S2. Missing data - absolute number of missing cases and missing percentage per outcome.

Variable	Absolute number of missing cases	Missing percentage
Visuomotor skills	1	1.4
Processing	1	1.4
Visual memory	1	1.4
Verbal memory	1	1.4
Visual working memory	1	1.4
Verbal working memory	1	1.4
Estimated_IQ	0	0
Internalizing problems (parent, SDQ)	0	0
Externalizing problems (parent, SDQ)	0	0
Internalizing problems (teacher, SDQ)	9	13
Externalizing problems (teacher, SDQ)	9	13
Attention problems (parent, SWAN)	3	4.3
Hyperactivity problems (parent. SWAN)	3	4.3
Attention problems (teacher, SWAN)	9	13
Hyperactivity problems (teacher, SWAN)	9	13
Arithmetic[1]	23	33.3
Spelling[1]	25	36.2
Technical reading[1]	23	33.3

[1] The missing data for the CITO test results for arithmetics, spelling, and technical reading were not imputed as more than 15% of the data was missing. This can have several reasons. Firstly, not all children attend schools that take part in regular CITO testing; not all parents were willing to share their child's test results; not all teachers and/or schools responded to our request to share CITO test results for the participant.
