# Supplementary material for: Neurodevelopmental outcomes of school-age children conceived after hysterosalpingography with oil-based or water-based iodinated contrast: long-term follow-up of a nationwide randomized controlled trial
Source: Hum Reprod. 2024 Aug 28;39(10):2287–96. doi: 10.1093/humrep/deae183 (PMC11447066; doi:10.1093/humrep/deae183)
Supplement: deae183_Supplementary_Table_S3 [file deae183_supplementary_table_s3.pdf]

Human Reproduction, 2024, 1
https://doi.org/10.1093/humrep/deae183
Supplementary Data

Supplementary Table S3. Checking for biases between the two study groups (water-based contrast versus oil-based contrast).

Variable	Oil (n = 42)	Water (n = 27)	Significance (*t*-test, chi-squared, Fisher exact test)
Age (mean ± SD)	7.65 ± 0.72	7.69 ± 0.77	0.819
Highest education parents (mean ± SD)	5.89 ± 0.79	6.09 ± 0.86	0.333
Sex (f%)	45.2%	63.0%	0.233
Smoking during pregnancy	9.5%	3.7%	0.642
Pregnancy complications	7.1%	11.1%	0.672
Postpartum complications	2.4%	3.7%	1
Congenital abnormalities	2.4%	0%	1

First, we checked whether the oil-based and water-based contrast groups were comparable on different demographic variables, looking at age, parental education (as a measure of socioeconomic status), and sex of the child. Henceforth, we have called the water-based iodinated contrast group ‘Water’ and the group of children born after their mother received HSG with oil-based iodinated contrast ‘Oil’. There are no significant differences between the two groups using $P < 0.05$. The groups are of similar age and sex and come from families with a similar composition of parental education. We have also checked for differences in pregnancy complications, postpartum complications, and congenital abnormalities but found no significant differences between the two contrast groups.
