# Supplementary material for: Neurodevelopmental outcomes of school-age children conceived after hysterosalpingography with oil-based or water-based iodinated contrast: long-term follow-up of a nationwide randomized controlled trial
Source: Hum Reprod. 2024 Aug 28;39(10):2287–96. doi: 10.1093/humrep/deae183 (PMC11447066; doi:10.1093/humrep/deae183)
Supplement: deae183_Supplementary_Table_S4 [file deae183_supplementary_table_s4.pdf]

OXFORD
human reproduction
Human Reproduction, 2024, 1
https://doi.org/10.1093/humrep/deae183
Supplementary Data

Supplementary Table S4. Checking for biases between the Neuro-H2Oil follow-up study and original H2Oil trial.

	H2Oil trial (2012–2014)	Neuro-H2Oil (2022)
Age of the mother at the time of HSG (median), age >35 years	32.9 years, 31.1%	32.0 years, 20.3%
BMI kg/m^2 (median), BMI > 30.0 kg/m^2 (%)	22.9 kg/m^2, 10.3%	22.4 kg/m^2, 7.7%
White[1]	74%	81%
Smoker at the time of HSG	16.4%	7.2%

The H2Oil trial was a randomized controlled trial conducted in 2012–2014 in locations throughout the Netherlands. For further details of the trial, we refer to the publication Dreyer et al. (2017). We wished to see if there was selection bias in the follow-up, however, there was no data from the H2Oil trial on socioeconomic status, occupation, or educational attainment to ensure a comparison of the demographic characteristics of the trial participants to the mothers whose children participated in the Neuro-H2Oil follow-up. Instead, we made use of characteristics that were collected in the H2Oil trial, necessitating a flexible approach to these limitations. We did not find any significant differences in key characteristics between the current sample and the original trial participants using $P < 0.05$.

[1] In the H2Oil trial, the variable 'race' was recorded as 'reported by the clinicians'. The Neuro-H2Oil trial did not actively collect data within such categorizations.
