# Supplementary material for: Neurodevelopmental outcomes of school-age children conceived after hysterosalpingography with oil-based or water-based iodinated contrast: long-term follow-up of a nationwide randomized controlled trial
Source: Hum Reprod. 2024 Aug 28;39(10):2287–96. doi: 10.1093/humrep/deae183 (PMC11447066; doi:10.1093/humrep/deae183)
Supplement: deae183_Supplementary_Table_S5 [file deae183_supplementary_table_s5.pdf]

human reproduction

Human Reproduction, 2024, 1
https://doi.org/10.1093/humrep/deae183
Supplementary Data

Supplementary Table S5. The neurocognitive outcomes of children conceived after oil- versus water-based iodinated contrast media use during HSG were corrected for age, sex, and parental educational attainment.

Domain	Oil-based contrast group (n = 42) M (SD)	Water-based contrast group (n = 27) M (SD)	Uncorrected mean difference	Adjusted mean difference (SE)	P-value	Confidence interval	
						Lower bound	Higher bound
Intelligence							
IQ	105.33 (11.23)	102.78 (12.61)	2.56	4.09 (2.81)	0.15	−1.53	9.71
Neurocognitive outcomes							
Visuomotor integration	**0.19 (0.89)**	**−0.31 (1.10)**	**0.50**	**0.46 (0.21)**	**0.03***	**0.04**	**0.89**
Processing and control	−0.01 (1.03)	0.02 (0.97)	−0.03	0.03 (0.22)	0.90	−0.42	0.48
Visual memory	0.12 (1.00)	−0.19 (0.98)	0.31	0.22 (0.24)	0.28	−0.69	0.62
Verbal memory	−0.05 (1.02)	0.08 (0.98)	−0.12	−0.17 (0.24)	0.48	−0.65	0.31
Visual working memory	0.09 (1.01)	−0.15 (0.99)	0.24	0.25 (0.23)	0.30	−0.22	0.71
Verbal working memory	0.00 (1.00)	0.00 (1.02)	0.00	0.12 (0.21)	0.59	−0.31	0.54
Behavioural outcome							
Internalizing problems (parent, SDQ)	3.76 (3.31)	2.78 (2.93)	0.98	0.82 (1.04)	0.30	−0.76	2.41
Externalizing problems (parent, SDQ	5.05 (4.67)	3.96 (2.86)	1.08	0.68 (1.03)	0.51	−1.37	2.73
Internalizing problems (teacher, SDQ)	1.90 (2.13)	2.33 (2.71)	−0.42	−0.25 (0.59)	0.67	−1.43	0.92
Externalizing problems (teacher, SDQ)	2.75 (2.95)	3.22 (2.76)	−0.47	−0.88 (0.71)	0.22	−2.29	0.53
Attention problems (parent, SWAN)	−0.34 (0.95)	−0.46 (0.68)	0.11	0.08 (0.22)	0.73	−0.37	0.52
Hyperactivity problems (parent, SWAN)	−0.26 (1.01)	−0.17 (0.65)	−0.09	−0.16 (0.23)	0.49	−0.61	0.29
Attention problems (teacher, SWAN)	−0.58 (0.89)	−0.48 (0.73)	−0.11	−0.14 (0.20)	0.48	−0.55	0.26
Hyperactivity problems (teacher, SWAN)	−0.53 (0.97)	−0.55 (0.93)	0.02	−0.06 (0.23)	0.78	−0.52	0.39
Academic performance[1]							
Arithmetic	0.74 (0.93)	0.31 (0.82)	0.44	0.43 (0.29)	0.13	−0.14	1.04
Spelling	0.56 (0.88)	0.64 (0.99)	−0.08	−0.14 (0.32)	0.74	−0.73	0.52
Technical reading	0.28 (0.99)	0.02 (1.02)	0.26	0.21 (0.32)	0.42	−0.43	0.86

Results for which $P < 0.05$ are shown in bold with an asterisk.
[1] Academic performance data are not imputed because >15% of the data are missing. The sample size of the oil-based contrast group is n = 32, and of the water-based contrast group is n = 14.

© The Author(s) 2024. Published by Oxford University Press on behalf of European Society of Human Reproduction and Embryology.
This is an Open Access article distributed under the terms of the Creative Commons Attribution License (https://creativecommons.org/licenses/by/4.0/), which permits unrestricted reuse, distribution, and reproduction in any medium, provided the original work is properly cited.
Human Reproduction, 2024, 00, 1–15
https://doi.org/10.1093/humrep/deae183
Original Article
